# Responses of corn physiology and yield to six agricultural practices over three years in middle Tennessee

**DOI:** 10.1038/srep27504

**Published:** 2016-06-07

**Authors:** Chih-Li Yu, Dafeng Hui, Qi Deng, Junming Wang, K. Chandra Reddy, Sam Dennis

**Affiliations:** 1 Department of Biological Sciences, Tennessee State University, Nashville, TN 37209, USA; 2 Climate and Atmospheric Science Section, Illinois State Water Survey, Prairie Research Institute, University of Illinois at Urbana-Champaign, Champaign, IL 61802, USA; 3 Department of Agricultural and Environmental Sciences, Tennessee State University, Nashville, TN 37209, USA

## Abstract

Different agricultural practices may have substantial impacts on crop physiology and yield. However, it is still not entirely clear how multiple agricultural practices such as tillage, biochar and different nutrient applications could influence corn physiology and yield. We conducted a three-year field experiment to study the responses of corn physiology, yield, and soil respiration to six different agricultural practices. The six treatments included conventional tillage (CT) or no tillage (NT), in combination with nitrogen type (URAN or chicken litter) and application method, biochar, or denitrification inhibitor. A randomized complete block design was applied with six replications. Leaf photosynthetic rate, transpiration, plant height, leaf area index (LAI), biomass, and yield were measured. Results showed that different agricultural practices had significant effects on plant leaf photosynthesis, transpiration, soil respiration, height, and yield, but not on LAI and biomass. The average corn yield in the NT-URAN was 10.03 ton/ha, 28.9% more than in the CT-URAN. Compared to the NT-URAN, the NT-biochar had lower soil respiration and similar yield. All variables measured showed remarkable variations among the three years. Our results indicated that no tillage treatment substantially increased corn yield, probably due to the preservation of soil moisture during drought periods.

Corn (*Zea mays*) is one of the most important grains in the United States^1^,^2^. On average, each American consumes 11.3 kg of corn annually. In 2012, around 97 million acres of land is used in the US to produce about 32% of the world’s corn crop^3^.Although global agricultural output has improved dramatically over the past 50 years, future demand for corn as food, feed, and bioenergy resource will increase tremendously due to the growing population^4^,^5^. The FAO report^6^ showed that annual cereal production will need to rise from the 2.1 billion today to about 3 billion tons in 2050^6^. Therefore, closing the yield gap has become a big challenge^4^. It is an urgent task for agricultural scientists and farmers to find the best agricultural practice and sustain corn productivity while improving environmental quality.

To improve corn growth and yield, and reduce greenhouse gas emission, various agricultural practices have been used, including the application of poultry litter, use of nitrification inhibitor or biochar, placement of nitrogen (N) fertilizer and split use of N at different growth stages^7^,^8^,^9^,^10^,^11^. In the U.S., an estimated 50.7 million tons of poultry litter was generated in 2007^12^,^13^. Poultry litter is a valuable resource that provides assorted plant nutrients and adds organic matter to the soil to improve soil fertility^7^. For example, the addition of poultry litter enhances corn yield, without changing corn biomass^7^. Efthimiadou *et al*.^14^ reported that combined organic/inorganic fertilizers contribute to higher crop productivity than mineral N fertilizer alone^14^. Nitrification inhibitors such as 3,4-dimethylpyrazole phosphate (DCD) are useful in not only delaying microbial nitrification and subsequently denitrification, but also in reducing N losses in the forms of nitrous oxide (N_2_O) emission and NO_3_^−^ leaching^15^,^16^. The application of DCD blended N enhances the yields of corn^17^, wheat^18^, and rice^19^. However, Weiske *et al*.^16^ found that DCD does not affect the yields of summer barley, maize, and winter wheat^16^. The split applications of N fertilizer may reduce N losses and improve yield and N use efficiency, but the effects vary with the application times and soil moisture^20^,^21^. Split N application in wheat can synchronize N supply with crop N demand and lead to a higher mean N recovery efficiency^22^. Similarly, a total of 170 kg N/ha applied in three splits is found to be more efficient than a single pre-planting application of 510 kg N/ha in corn^23^. But Liu *et al*.^24^ found that split N application has no effect on corn yield and plant characteristics in dry-land conventional tillage system, because of water stress during the reproductive stage^24^. Therefore, responses of crop physiology and yield may vary among different crop systems and weather conditions.

In most countries, farmers still use a tillage system before seeding to control for weeds and pests. However, compared to conventional tillage, no-till systems have been shown to improve soil quality by increasing organic matter and infiltration, and reducing input costs, greenhouse emissions and soil erosion^25^,^26^. DeFelice *et al*.^27^ reported that the effect of tillage may vary with climate factors and regions^27^; corn yield with conventional tillage is higher than no-tillage in the northern regions of the U.S. and Canada, lower in southern and western regions, and corn yield using the two methods are similar in the central regions^27^.

Application of biochar may improve grain yield and reduce soil CO_2_ emission (i.e. soil respiration)^28^,^29^,^30^. A meta-analysis showed the effect of biochar on crop yield ranges from −28% to +39%, with a grand mean of increase by 10%^31^. The effects of biochar on the yields of rice and sorghum are often positive^32^, but negative on ryegrass^33^. The effects of biochar on corn growth and yield vary with biochar applications^34^,^35^. Uzoma *et al*.^36^ reported that corn yield and water use efficiency (WUE) are significantly increased by applying 15 or 20 ton ⁄ ha of biochar^36^. However, corn yield under the application of combined biochar/inorganic N fertilizer has no significant effect, compared to the application of N fertilizer alone^9^. A comprehensive study of different agricultural practices on corn physiology, growth and yield over multiple years in the Southeastern U.S. is still needed.

A three-year field experiment was conducted to determine the effects of different agricultural practices on corn growth, photosynthesis and yield in middle Tennessee. The main objective of this study was to understand how agricultural practices could influence corn physiological performance, biomass and yield in different years. Specifically, we tested 1) how different agricultural practices influenced corn physiology, growth and yield? 2) whether the effects of the agricultural practices varied among the three years? and 3) what was the best agricultural practice for obtaining a high yield and maintaining a low soil respiration? The information generated in this study will be helpful to select the best agricultural practice that can produce a high yield and maintain a low soil respiration.

## Results

### Significance tests of treatment, year and their interactions

Analysis of Variance (ANOVA) results showed that treatment had significant effects on plant physiology (p < 0.0001 for leaf photosynthesis, transpiration, and WUE; p = 0.0017 for stomatal conductance), soil respiration (p < 0.0001), height (p = 0.0073), and yield (p = 0.0281), but not on leaf area index (LAI), above- and below-ground biomass, and root:shoot (R:S) ratio (Tables 1 and 2). Significant differences in all variables among years were found at α = 0.01 level. For plant physiology and soil respiration, significant differences were also found among different growth stages. Significant interactions between treatment and year were only found on transpiration (p = 0.0003) and LAI (p = 0.0397).

Among all treatments, the NT-litter (no-tillage + 20% N from URAN + 80% N from chicken litter) had the highest photosynthetic rate, stomatal conductance and WUE, and the CT-URAN (conventional tillage + regular application of 100% N from URAN) had the lowest photosynthesis and stomatal conductance (Table 3). The NT-biochar (no-tillage + regular applications of 100% N from URAN + woodchips biochar) had the lowest WUE. For soil respiration, the CT-URAN and NT-biochar had the lowest values. The NT-inhibitor (no-tillage +regular applications of 90% N from +10% N from dicyandiamide (DCD) nitrification inhibitor) had the highest LAI and the NT-split (no-tillage + 4 split applications of 100% N from URAN) and NT-litter had the lowest values. The tallest plants were found in the NT-litter and the shortest in the NT-split. Yield in the NT-litter and NT-URAN (no-tillage + regular applications of 100% N from URAN) had the highest values and the CT-URAN had the lowest yield.

To illustrate the effects of different agricultural practices, the effects of nitrogen application treatments, biochar, tillage and denitrification inhibitor treatment are described separately below. As no significant differences in above- and below-ground biomass and R:S ratio were found among all treatments, the biomass and R:S ratio were not described further.

### Effects of nitrogen fertilizer type and application time

Compared to the NT-URAN, the corn plants in the NT-Litter treatment had higher values in the photosynthetic rate (36.09 μmol CO_2_/m^2^/s), stomatal conductance (0.357 mol H_2_O/m^2^/s) and WUE (5.22 μmol CO_2_/mmol H_2_O), but no differences in transpiration, LAI, height and yield. The NT-split treatment significantly enhanced leaf photosynthesis (35.56 μmol CO_2_/m^2^/s) and stomatal conductance (0.356 mol H_2_O/m^2^/s) at α = 0.05 level, but did not change leaf transpiration, WUE, LAI, height and yield, compared to the NT-URAN.

### Effects of tillage, biochar and denitrification inhibitor

We compared CT-URAN and NT-URAN for the tillage effects. Over the three years, the CT-URAN had lower photosynthetic rate (32.60 μmol CO_2_/m^2^/s), stomatal conductance (0.319 mol H_2_O/m^2^/s), transpiration (7.14 mmol H_2_O/m^2^/s), soil respiration (3.09 μmol CO_2_/m^2^/s) and yield (7.78 ton/ha), compared to the NT-URAN (Table 2). No differences in WUE, LAI, and height were found between the two treatments.

Compared to the NT-URAN, the stomatal conductance (0.355 mol H_2_O/m^2^/s) and transpiration (7.92 mmol H_2_O/m^2^/s) of corn plants in the NT-biochar were significantly higher at α = 0.05 level. There were no significant differences in other variables between the NT-biochar and NT-URAN treatments.

In the NT-inhibitor, WUE (4.83 μmolCO_2_/m^2^/s) was significantly higher than the NT-URAN. There were no differences on other variables.

### Variations among the three years

Significant differences for leaf physiology (p < 0.0001 for leaf photosynthesis, stomatal conductance, transpiration, and WUE), growth (p < 0.0001 for above-ground, below-ground, and total biomass, height, and LAI; p = 0.0006 for R:S ratio), soil respiration (p < 0.0001) and yield (p < 0.0001) were observed among the 3 years (Tables 1, 2 and 3). The mean leaf photosynthetic rate, stomatal conductance, transpiration and plant height in 2013 were significantly higher than in the other two years, but LAI was lower (Table 4). WUE and biomass in 2014 were higher than in the other two years. Yield in 2014 was the highest (13.42 ton/ha) and almost more than doubled that in 2012 (6.03 ton/ha).

### Interactions between treatment and year

**Figure 1:**
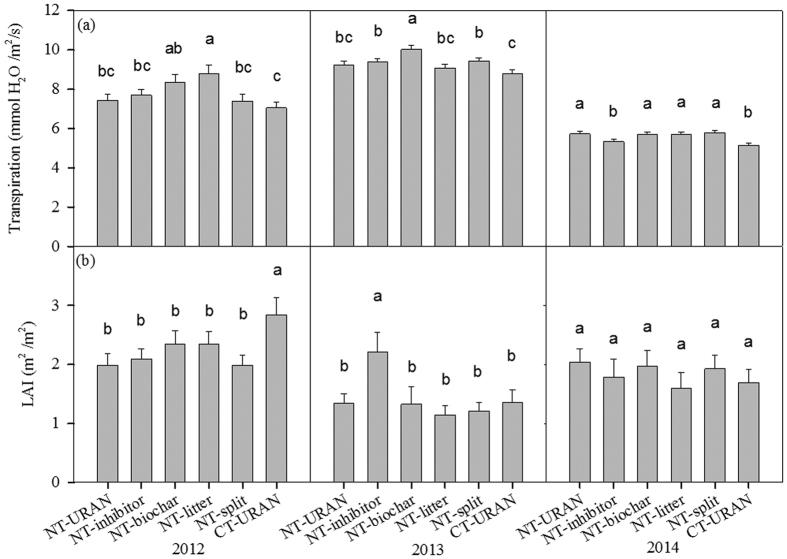
Interactive effects of treatment and year on transpiration (**a**) and leaf area index (LAI, **b**) among 3 years.

The interactive effect of treatment and year was significant on transpiration (p = 0.0003) and LAI (p = 0.0397), but not on leaf photosynthesis rate or yield (Tables 1 and 2). Transpiration in the NT-biochar was among the highest values in three years (Fig. 1a). LAI in the CT-URAN was significantly higher in 2012 and 2014 at α = 0.05 level, but in 2013, LAI in the NT-inhibitor was the highest (Fig. 1b).

### Variation of leaf physiological variables among corn plant growth stages

**Figure 2:**
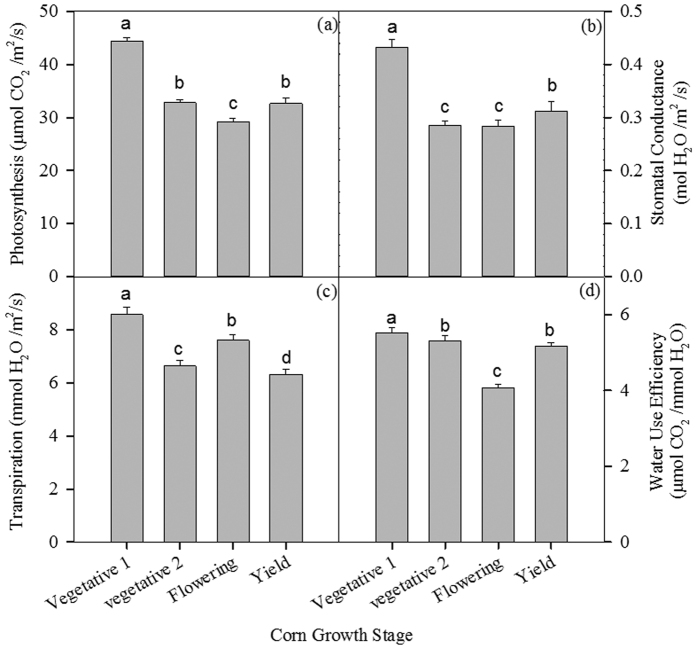
Mean and significance of leaf photosynthesis rate (**a**), stomatal conductance (**b**), transpiration (**c**), water use efficiency (WUE, **d**), and soil respiration (**e**) of corn in different growth periods among 3 years.

Significant differences for leaf photosynthesis (p < 0.0001), stomatal conductance (p < 0.0001), transpiration (p < 0.0001) and WUE (p < 0.0001) were observed among different corn growth stages (Table 1). Plants at the Vegetative 1 Stage had the highest leaf photosynthesis, stomatal conductance, transpiration and WUE (Fig. 2a–d). Plants at the Flowering Stage had the lowest leaf photosynthesis, stomatal conductance and WUE.

### Relationships between plant growth and leaf physiological variables

**Figure 3:**
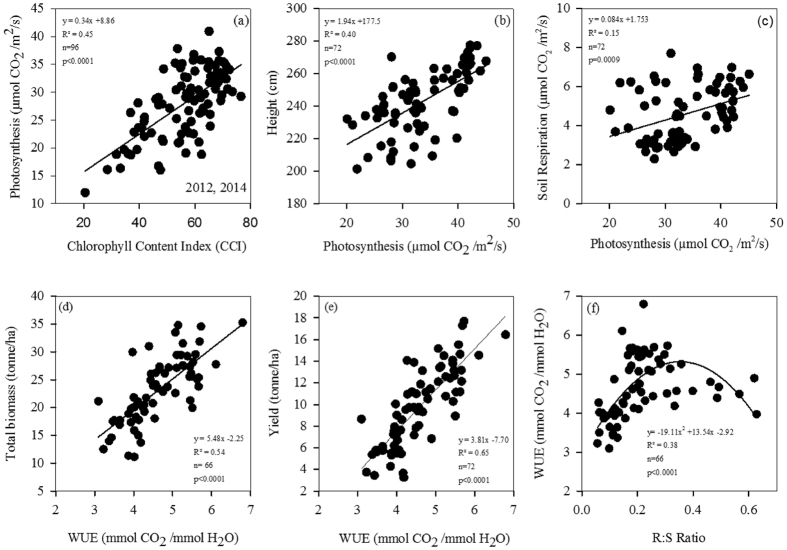
Relationships of the leaf photosynthesis rate and chlorophyll content index (CCI, **a**), plant height (**b**), soil respiration (**c**), of water use efficiency (WUE) with total biomass (**d**), yield (**e**), and root:shoot (R:S) ratio (**f**).

Regression analysis revealed that the leaf photosynthesis rate was positively correlated with leaf chlorophyll content (CCI) (Fig. 3a). Plant height and soil respiration linearly increased with leaf photosynthesis (Fig. 3b,c). Plants grew taller when plants had higher leaf photosynthesis rate. High leaf photosynthesis rate also enhanced soil respiration. Corn yield and biomass linearly increased with WUE (Fig. 3d,e). WUE increased with R:S ratio, reached the highest value when R:S ratio was about 0.35, and decreased as R:S ratio increased (Fig. 3f).

## Discussion

### Effects of fertilizer type and application time

In this study, the same amount of nitrogen was applied to all treatments, but with different fertilizer types and application methods. Compared to typical agricultural practices used by farmers in middle Tennessee (the NT-URAN treatment), using poultry litter (the NT-litter) and split application of nitrogen fertilizer (the NT-split) enhanced corn leaf photosynthesis, stomatal conductance and soil respiration, but did not increase plant growth (LAI and height) and yield. Our results were different from some previous studies that reported higher crop yield with poultry litter application. For example, Khaliq *et al*.^37^ found that corn yield after fertilization with 200 N of poultry litter (5.29 ton/ha) or a half of poultry litter plus a half of urea (5.98 ton/ha) was significantly higher than that fertilized with all urea (4.43 ton/ha)^37^. Endale *et al*.^7^ also showed that average yield with poultry litter (7.77 ton/ha) application was higher than that with ammonium nitrate/sulfate (6.57 ton/ha) application in a 5 years field experiment, but above-ground biomass was not influenced^7^.One possible reason was that the amount of chicken litter applied in our study was lower than that used in these previous studies and it might take a longer time before chicken litter would show its effects on crop growth and yields. We did find leaf photosynthesis to be higher in the NT-litter treatment, similar to Efthimiadou *et al*.^14^ who reported that leaf photosynthesis of sweet corn fertilized by poultry manure (140 kg N/ha) and ammonium sulphate (100 kg N/ha) was higher than ammonium sulphate (240 kg N/ha). The effects of nitrogen on plant growth could also be influenced by other factors such as soil moisture conditions and other nutrients such as phosphorus.

Several researchers showed that split applications of N fertilizer often result in higher yield and nitrogen use efficiency. The N applied by pre-planting and side-dressing at the critical growth stages can increase the corn yield^38^,^39^. In this study and similar to some of the other researchers’ split application, N fertilizer was applied twice (at jointing stage and heading stage), as in the typical N applications here. However, we did not find a significant change in leaf physiology, growth and yield in the NT-split, compared to the NT- URAN. The main reason could be the N fertilizer applied in the typical N fertilizer application treatments during the growth period could support enough N demand for corn growth. Even though we increased the times of N application in the NT-split, the total amount of the N fertilizer applied was the same among all treatments. Thus, no difference of corn growth and yield was observed.

### Effects of tillage, biochar, and nitrification inhibitor

Consistent with other studies, our results showed that the NT-URAN significantly increased leaf photosynthesis and corn yield compared to the CT-URAN at α = 0.05 level. For example, Endale *et al*.^7^ showed corn yield in the NT treatment (7.56 ton/ha) is significantly higher than in the CT treatment (6.79 ton/ha)^7^. In another study, Karunatilake *et al*.^40^ reported that the average corn yield (7.26 ton/ha) from 1993 to 1999 in non-tillage is higher than in spring plow tillage (6.42 ton/ha)^40^. The major reason could be that the CT treatment decreased soil bulk density and soil moisture content, and as a result, decreased corn root growth and yield^41^,^42^. There are also a few studies reporting that no-tillage does not change or even decreases crop yield^40^,^43^. For example, Guan *et al*.^44^ reported that corn yields in the NT in 2011 and 2012 (6.76 and 9.89 ton/ha) are lower than in the CT (7.08 and 10.81 ton/ha) in the North China Plain^44^. They attributed this to poor root growth in the NT^43^. In this study, soil respiration in the NT treatment was higher than in the CT treatment, partially due to high soil moisture in the NT treatment^45^,^46^. The higher photosynthesis in the NT treatment also contributed to the higher soil respiration (Table 4, Fig. 3c). While soil CO_2_ emission is often enhanced by the NT treatment, soil carbon sequestration could be higher due to more carbon inputs through photosynthesis and growth, as demonstrated by a recent meta-analysis in Mediterranean cropping systems^47^.

We did not find significant treatment effects in the NT-biochar treatment on most of the variables measured except soil respiration which was substantially decreased. Different from Zhang’s research, the application of biochar in the corn field increased soil respiration and yield, probably due to the differences in soil texture or climate^48^. Gaskin *et al*. also did not find a significant difference in corn yield when biochar was applied at 0, 11, and 22 Mg/ha with N fertilizer^9^. Similarly, Major *et al*.^35^ found corn yield does not change the first year when treated with 8 or 20 ton/ha of biochar, but increases in the following three years, probably due to biochar improvement of soil properties^35^. Positive effects of biochar on corn yield have been reported in several other studies^35^,^49^. Martinsen *et al*. found corn yield benefits from biochar application when plots are sufficiently irrigated^49^. In our study, a drought year of 2012 when biochar was applied could influence the effect of biochar on corn physiology and growth.

Similar to some prior studies, NT-inhibitor treatment did not influence plant biomass and yield^10^,^15^. This is reasonable as the same total amount of N was applied to all treatments. While corn yield was not influenced by the NT-inhibitor, N_2_O emissions were reduced^46^. Soil respiration was not significantly influenced.

### Variation among three years

Due to remarkable differences in precipitation intensity and pattern among the three years, all variables we measured in this study showed significant differences among years at α = 0.05 level. The mean leaf photosynthesis rate in 2013 was much higher than in 2012 or 2014 (Table 3). In 2012, little rainfall was received during the flowering period in June. Even though the corn plots were irrigated twice, the severe drought in 2012 decreased leaf photosynthesis and WUE, which resulted in lower biomass and yield. R:S ratio was also lower in 2012 than other two years, probably due to the severe drought caused more death to fine roots and reduced root biomass. Cakir^50^ found that the corn yield is strongly decreased by prolonged water stress during the tasselling and ear formation stages^50^.There was relatively more rainfall during the tasselling and ear formation stages in 2013 and 2014, resulting in higher yields than in 2012.

Precipitation change among years may also affect the treatment effects. But in this study, only transpiration and LAI had significant interactive effects of treatment and year. This could be due to that we only had one treatment level for some treatment factors such as biochar and DCD inhibitor. Further studies with multiple treatment factors and treatment levels need to be conducted.

### Controls of leaf physiology, plant growth and yield

Leaf photosynthetic rate is often correlated to the quantity of chlorophyll content in the leaf 20,51,52. Our result confirmed that there was a positive linear relationship between leaf photosynthesis and CCI (Fig. 3a). The relationship between yield and photosynthesis is still not conclusive. Heichel and Musgrave (1969) showed the relationship between photosynthesis and yield in corn is poor^53^, while Edmeades and Daynard (1979) reported significant nonlinear relationships of photosynthesis with corn yield and shoot biomass^54^. We found that plant height seemed to linearly increase with photosynthesis.

The prior studies indicated higher leaf photosynthesis wasn’t equal to higher crop yield production^55^. Therefore, leaf WUE could be another factor to evaluate the aboveground biomass or crop yield, but in a lot of times no relationship was shown^56^. In this study, WUE was strongly correlated to total biomass (R^2^ =  0.68) or yield (R^2^ =  0.65), indicating that improving corn WUE has a potential to increase corn yield.

## Conclusion

In this three-year field experiment, we found that different nitrogen fertilizers and application methods did not significantly influence corn yield, even though the management practices influenced leaf physiology and growth. No tillage treatment substantially increased corn yield by preserving soil moisture during drought summers in Nashville. Leaf photosynthesis could influence corn yield. All variables measured showed significant differences among years, indicating that interannual variations in climatic factors could have considerable influences on plant growth and yield stability. Further studies on the optimal amount of poultry litter and optimal time for irrigation are needed to establish a sustainable high corn production system.

## Methods

### Site description

The field experiment was conducted from 2012–2014 at the Tennessee State University Agricultural Research Center (Latitude 36.12′N, Longitude 36.98′W, elevation 127.6 m) in Nashville, Tennessee, USA^46^. Nashville has a humid subtropical climate with hot summers. Rainfall is typically greater in November and December, and the spring, while August to October is the driest period in general^57^. The soil is Talbott silt clay loam (Fine, mixed, semi-active, thermic Typic Hapludalfs; 25% sand, 55% silt, 20% clay), slightly acidic (pH  =  5.97), low in both carbon (2.37 g/kg) and nitrogen (0.14 g/kg).

### Experimental Design

A randomized complete block design was applied with six treatments and six replications. Six treatments were randomly assigned to each plot in one block. Referring to the common practice by farmers in middle Tennessee, we considered the treatment with conventional tillage +regular applications of aqueous urea ammonium nitrate (URAN-32-0-0 liquid N, 100% N from URAN-32-0-0) as the control (CT-URAN). No-tillage and improved fertilizer management were used as the other five treatments: NT-URAN, no-tillage + regular applications of URAN (100%); NT-inhibitor, no-tillage + regular applications of URAN (90% N from URAN) + dicyandiamide (DCD) nitrification inhibitor (N, 10% N from the inhibitor); NT-biochar, no-tillage + regular applications of URAN (100%) + woodchips biochar; NT-litter, no-tillage + 20% applications of URAN (20% N from URAN) + chicken litter (N, 80% N from the litter); and NT-split, no-tillage + split applications of URAN (100%). The biochar was manufactured by Western Biochar LLC (Niwot, CO, USA) with a density of 1.5–1.7 g cm^−3^, and was applied at a rate of 2.5 kg m^−2^. The URAN-32-0-0 liquid N was purchased from a local nutrient company in Nashville. The plot size was 5.5 m × 7.0 m. Biochar was mixed in the top soil on April 23, 2012. Corn seeds (Roundup Ready BT Hybrid Corn, P1412 HR, Pioneer Hi-Bred International Inc., Johnston, IA) were planted at 100,500 seeds/ha on Apr 9, 2012, Apr 25, 2013 and May 8, 2014. Corn was planted at 0.5 m plant interval and 12 rows per plot at a density of 100,500 plant/ha. Biochar was applied once before the seeding in the NT-biochar plots in 2012, and not applied in the next two years.

The total nitrogen amount applied in each plot was 217 kg N/ha. At first, the N fertilizer (99 kg N/ha) was spread after corn seeding. During the growing period, urea fertilizer was applied twice in each plot at jointing stage (39 kg N/ha) and heading stage (79 kg N/ha), excluding the NT-split plots. The plots under the NT-split treatments were spread by two additional fertilizer applications of 19.5 and 39.5 kg N/ha (4 fertilizer applications in total) before tasseling stage. Due to the severe drought in June, 2012 that dramatically influenced plant growth, we irrigated all plots on June 14–15 at an equivalent of 50 mm water and June 30-July 2 at an equivalent of 90 mm. No irrigation was conducted in 2013 and 2014.

### Field measurements of corn physiology, soil respiration, plant growth, and yield

Leaf photosynthesis, stomatal conductance, and transpiration were measured with a Li-6400 Portable Photosynthesis System (Li-Cor Inc., Lincoln, NE, USA) four times during the growing season at 35–42 days after planting (DAP) (Vegetative stage 1), 57–64 DAP (Vegetative stage 2), 75–79 DAP (Flowering stage), and 90–101 DAP (Yield stage). Two fully expanded young leaves were measured from two to four randomly selected plants in the middle of a plot between 10:00am and 3:00pm during the growing period. The leaf photosynthesis measurement was set at 2000 μmol photon/m^2^/s for photosynthetically active radiation, and 380~400 ppm for ambient CO_2_ concentration. Leaf WUE was calculated as leaf photosynthesis/ transpiration. Chlorophyll content index (CCI) was measured using a CCM-200 plus (Opti-Sciences, Inc., Hudson, NH, USA) after leaf photosynthesis measurement at the same time. LAI of each plot was measured at the flowering period each year using an LAI 2200 Plant Canopy Analyzer (Li-Cor Inc., Lincoln, NE, USA). Soil respiration was measured using static chambers during the growing season. The construction of the static chambers, gas sampling and calculation are described in detail in Deng *et al*.^46^.

The plant height was measured before harvesting. Corn grain yields and total biomass were harvested from the middle two rows (each 2.4 m long) in each plot, separately. Corn ears were removed by hand after harvesting, then shelled and dried to 14% moisture. Prior to shelling, ear type categories of incomplete, complete, blunt, of nubbin were determined using the method proposed by Mueller and Pope^58^. Above- and below-ground biomass was measured after harvesting. Whole plants were weighed and then chopped with a saw to facilitate sub-sampling. Subsamples were weighed and then oven dried at 50 °C before being reweighed to determine water content.

### Statistical analysis

Data analysis was performed using SAS software 9.1^59^. The effects of treatment, year and their interactions were analyzed using ANOVA. Due to weeds problems in two blocks in 2014, only four blocks were used in data analysis for 2014. In 2012, leaf photosynthetic rates were missing during the vegetative stages. PROC GLM was used for ANOVA. When a significant effect was detected, least significant difference (LSD) method was used for multiple comparison. Regression analysis was conducted to detect whether plant growth, yield and soil respiration were related to leaf photosynthesis and WUE. Significant level was mostly set at α = 0.05.

## Additional Information

**How to cite this article**: Yu, C.-L. *et al*. Responses of corn physiology and yield to six agricultural practices over three years in middle Tennessee. *Sci. Rep*. **6**, 27504; doi: 10.1038/srep27504 (2016).

## Figures and Tables

**Table 1:** Significance of the effects of treatment, year, growth stage, and interaction between year and treatment on corn leaf physiology and soil respiration using ANOVA.

Source	Leaf Photosynthesis (μmol CO_2_/m^2^/s)	Stomatal Conductance (mol H_2_O/m^2^/s)	Transpiration (mmol H_2_O/m^2^/s)	WUE (μmol CO_2_/mmol H_2_O)	Soil Respiration (μmol CO_2_/m^2^/s)
Block	13.04^**^	0.72	16.68^**^	19.81^**^	6.09^**^
Growth Stage	479.69^**^	581.94^**^	1134.12^**^	386.12^**^	^–^
Year	252.07^**^	105.53^**^	141.40^**^	145.37^**^	49.32^**^
Treatment	7.22^**^	3.88^**^	10.05^**^	9.49^**^	6.18^**^
Treatment × Year	1.28	1.38	3.31^**^	1.35	1.02

Numbers are F-values. Stars indicate the level of significance (^*^ = *p* < 0.05, ^**^ = *p* < 0.01).

**Table 2:** Significance of the effects of treatment, year and their interactions on corn growth and yield using ANOVA.

Source	LAI (m^2^/m^2^)	Height (cm)	Above-ground Biomass (ton/ha)	Root Biomass (ton/ha)	Total Biomass (ton/ha)	R:S ratio	Yield (ton/ha)
Block	2.67^**^	2.96^*^	3.09^*^	3.19^**^	3.47^*^	2.48	3.80^**^
Year	22.98^**^	19.70^**^	37.42^**^	24.13^**^	44.45^**^	18.66^**^	95.73^**^
Treatment	1.47	3.46^**^	1.06	1.11	0.88	1.30	2.68^*^
Treatment × year	2.04^*^	1.66	1.28	0.97	1.41	0.75	0.50

Numbers are F-values. Stars indicate the level of significance (^*^ = *p* < 0.05, ^**^ = *p* < 0.01).

**Table 3:** Multiple comparisons of leaf physiology, growth, and yield of corn under different tillage and fertilizer treatments.

Treatment	Photosynthesis (μmol CO_2_/m^2^/s)	Stomatal Conductance (mol H_2_O/m^2^/s)	Transpiration (mmol H_2_O/m^2^/s)	WUE (μmol CO_2_/mmol H_2_O)	Soil Respiration (μmol CO_2_/m^2^/s)	LAI (m^2^/m^2^)
NT-URAN	34.50 ± 1.57^b^	0.334 ± 0.025^b^	7.67 ± 0.39^bc^	4.70 ± 0.18^cd^	3.49 ± 0.11^a^	1.72 ± 0.15^ab^
NT-inhibitor	34.19 ± 1.58^b^	0.331 ± 0.026^b^	7.50 ± 0.41^c^	4.83 ± 0.21^b^	3.59 ± 0.12^a^	2.07 ± 0.20^a^
NT-biochar	35.06 ± 1.79^ab^	0.355 ± 0.030^a^	7.92 ± 0.44^a^	4.61 ± 0.18^d^	3.19 ± 0.10^b^	1.87 ± 0.22^ab^
NT-litter	36.09 ± 1.52^a^	0.357 ± 0.027^a^	7.66 ± 0.41^bc^	5.00 ± 0.19^a^	3.73 ± 0.12^a^	1.67 ± 0.20^b^
NT-split	35.56 ± 1.68^a^	0.356 ± 0.028^a^	7.87 ± 0.41^ab^	4.70 ± 0.18^cd^	3.65 ± 0.12^a^	1.65 ± 0.16^b^
CT-URAN	32.60 ± 1.74^c^	0.319 ± 0.028^b^	7.14 ± 0.42^d^	4.76 ± 0.18^bc^	3.09 ± 0.10^b^	1.95 ± 0.25^ab^
Treatment	Height (cm)	Above-ground Biomass (ton/ha)	Root Biomass (ton/ha)	Total Biomass (ton/ha)	R:S ratio	Yield (ton/ha)
NT-URAN	239.60 ± 5.67^ab^	19.67 ± 1.24^a^	4.74 ± 0.93^a^	24.41 ± 1.94^a^	0.23 ± 0.04^ab^	10.03 ± 0.92^a^
NT-inhibitor	243.85 ± 6.39^a^	19.46 ± 1.56^a^	4.01 ± 0.52^a^	23.47 ± 2.01^a^	0.20 ± 0.02^ab^	9.69 ± 1.14^a^
NT-biochar	243.19 ± 4.64^a^	19.33 ± 1.48^a^	4.33 ± 0.91^a^	23.66 ± 2.04^a^	0.22 ± 0.05^ab^	9.10 ± 1.18^ab^
NT-litter	251.11 ± 4.21^a^	19.61 ± 1.18^a^	4.50 ± 0.74^a^	24.11 ± 1.46^a^	0.24 ± 0.05^ab^	10.10 ± 1.09^a^
NT-split	230.52 ± 6.66^b^	18.68 ± 1.02^a^	3.17 ± 0.43^a^	21.85 ± 2.96^a^	0.16 ± 0.02^b^	9.55 ± 0.92^a^
CT-URAN	247.45 ± 4.62^a^	17.38 ± 0.93^a^	4.75 ± 0.81^a^	22.13 ± 2.85^a^	0.27 ± 0.04^a^	7.78 ± 0.94^b^

Numbers are means  ±  standard errors. Different letters in the same column indicate statistical significance at *α* = 0.05. Sample size for photosynthesis, stomatal conductance, transpiration and WUE is 44, for soil respiration is 105, and for other variables is 12. The treatments include: NT-URAN = no-tillage + regular applications of URAN; NT-inhibitor = no-tillage + regular applications of URAN + nitrification inhibitor; NT-biochar = no-tillage + regular applications of URAN + biochar; NT-litter = no-tillage + chicken litter; NT-split = no-tillage + split applications of URAN; and CT-URAN = conventional tillage + regular applications of URAN.

**Table 4:** Mean and significance of leaf physiological performance, height and yield of corn in three years.

Year	Photosynthesis (μmol CO_2_/m^2^/s)	Stomatal Conductance (mol H_2_O/m^2^/s)	Transpiration (mmol H_2_O/m^2^/s)	WUE (μmol CO_2_/mmol H_2_O)	Soil Respiration (μmol CO_2_/m^2^/s)	LAI (m^2^/m^2^)	Height (cm)	Total Biomass (ton/ha)	R:S Ratio	Yield (ton/ha)
2012	28.86c	0.23c	7.75b	3.57c	3.78^a^	2.45a	235.94b	16.76c	0.10c	6.03c
2013	40.97a	0.45a	9.33a	4.44b	2.89^b^	1.38c	257.16a	23.29b	0.30a	10.01b
2014	30.03b	0.25b	5.57c	5.50a	2.99^b^	1.80b	234.76b	28.13a	0.23b	13.42a

Different letters in the same column indicate statistical significance at *α = *0.05. Sample size for photosynthesis, stomatal conductance, transpiration and WUE is 88, for soil respiration is 210, and for other variables is 24.

## References

[b1] Duvick D. N. The contribution of breeding to yield advances in maize (*Zea mays* L.). Adv. Agron. 86, 83–145 (2005).

[b2] Huang H. . Nitrous oxide emissions from a commercial cornfield (*Zea mays*) measured using the eddy-covariance technique. Atmos. Chem. Phys. 14, 12839–12854 (2014).

[b3] NCGA, The World of Corn: Unlimited Possibilities. Available at: http://www.ncga.com/upload/files/documents/pdf/WOC%202013.pdf. (Date of access: 01/03/2016) (2013).

[b4] Godfray H. C. J. . Food security: the challenge of feeding 9 billion people. Science. 327, 812–818 (2010). 20110467 10.1126/science.1185383

[b5] Tilman D., Cassman K. G., Matson P. A., Naylor R. & Polasky S. Agricultural sustainability and intensive production practices. Nature. 418, 671–677 (2002). 12167873 10.1038/nature01014

[b6] FAO, How to Feed World in 2050. Available at: http://www.fao.org/fileadmin/templates/wsfs/docs/expert_paper/How_to_Feed_the_World_in_2050.pdf. (Date of access: 01/03/2016) (2009).

[b7] Endale D. M. . No-till corn productivity in a Southeastern United States ultisol amended with poultry Litter. Agron. J. 100, 1401–1408 (2008).

[b8] Fox R. H., Kern J. M. & Piekielek W. P. Nitrogen fertilizer source, and method and time of application effects on no-till corn yields and nitrogen uptakes. Agron. J. 78, 741–746 (1986).

[b9] Gaskin J. W. . Effect of peanut hull and pine chip biochar on soil nutrients, corn nutrient status, and yield. Agron. J. 102, 623–633 (2010).

[b10] Liu C., Wang K. & Zheng X. Effects of nitrification inhibitors (DCD and DMPP) on nitrous oxide emission, crop yield and nitrogen uptake in a wheat-maize cropping system. Biogeosciences 10, 2427–2437 (2013).

[b11] Vetsch J. A. & Randall G. W. Corn production as affected by nitrogen application timing and tillage. Agron. J. 96, 502–509 (2004).

[b12] Ritz C. W. & Merka W. C. Maximizing poultry manure use through nutrient management planning. University of Georgia Cooperative Extension Bulletin, 1245 (2009).

[b13] USDA. 2007 Census of agriculture. Available at: http://www.agcensus.usda.gov/Publications/2007. (Date of access: 01/03/2016) (2009).

[b14] Efthimiadou A., Bilalis D., Karkanis A., Froud-Williams B. & Eleftherochorinos I. Effects of cultural system (organic and conventional) on growth, photosynthesis and yield components of sweet corn (*Zea mays* L.) under semi-arid environment. Not. Bot. HortiAgrobot. Cluj-Napoca. 37, 104–111 (2009).

[b15] Kelliher F. M., Clough T. J., Clark H., Rys G. & Sedcole J. R. The temperature dependence of dicyandiamide (DCD) degradation in soils: a data synthesis. Soil Biol. Biochem. 40, 1878–1882 (2008).

[b16] Weiske A., Benckiser G. & Ottow J. C. G. Effect of the new nitrification inhibitor DMPP in comparison to DCD on nitrous oxide (N_2_O) emissions and methane (CH_4_) oxidation during 3 years of repeated applications in field experiments. Nut. Cycl. Agroecosys. 60, 57–64 (2001).

[b17] Sharma S. N. & Prasad R. Use of nitrification inhibitors (neem and DCD) to increase N efficiency in maize-wheat cropping system. Fert. Res. 44, 169–175 (1995).

[b18] Sharma S. N. & Kumar R. Effects of dicyandiamide (DCD) blended with urea on growth, yield and nutrient uptake of wheat. J. Agr. Sci. 131, 389–394 (1998.)

[b19] Wells B. R. . Dicyandiamide (DCD) as a nitrification inhibitor for rice culture in the United States. Commun. Soil Sci. Plan. 20, 2023–2047 (1989).

[b20] Buttery B. R. & Buzzell R. I. The relationship between chlorophyll content and rate of photosynthesis in soybeans. Can. J. Plant Sci. 57, 1–5 (1977).

[b21] Feinerman E., Choi E. K. & Johnson S. R. Uncertainty and split nitrogen application in corn production. Am. J. Agric. Econ. 72, 975–984 (1990).

[b22] Riley W. J., Ortiz-Monasterio I. & Matson P. A. Nitrogen leaching and soil nitrate, nitrite, and ammonium levels under irrigated wheat in Northern Mexico. Nutr. Cycl. Agroecosys. 61, 223–236 (2003).

[b23] Fernández J. E., Murillo J. M., Moreno F., Cabrera F. & Fernández-Boy E. Reducing fertilization for maize in southwest Spain. Commun. Soil Sci. Plant Anal. 29, 2829–2840 (1998).

[b24] Liu K. & Wiatrak P. Corn production and plant characteristics response to N fertilization management in dry-land conventional tillage system. Int. J. Plant Prod. 5, 405–416 (2011).

[b25] Horowitz J., Ebel R. & Ueda K. “No-till” farming is a growing practice. Economic Information Bulletin 70, 28 (2010).

[b26] Uri N. D. Perceptions on the use of no-till farming in production agriculture in the United States: an analysis of survey results. Agric. Ecosyst. Environ. 77, 263–266 (2000).

[b27] DeFelice M. S., Carter P. R. & Mitchell S. B. Influence of tillage on corn and soybean yield in the United States and Canada. Crop Manage. 5, doi: 10.1094/CM-2006-0626-01-RS (2006).

[b28] Cornelissen G. . Biochar effect on maize yield and soil characteristics in five conservation farming sites in Zambia. Agron. 3, 256–274 (2013).

[b29] Harris K., Gaskin J., Cabrera M., Miller W. & Das K. C. Characterization and mineralization rates of low temperature peanut hull and pine chip biochars. Agron. 3, 294–312 (2013).

[b30] Lentz R. D. & Ippolito J. A. Biochar and manure affect calcareous soil and corn silage nutrient concentrations and uptake. J. Environ. Qual. 41, 1033–1043 (2012). 22751045 10.2134/jeq2011.0126

[b31] Jeffery S., Verheijen F. G. A., van der Velde M. & Bastos A. C. A quantitative review of the effects of biochar application to soils on crop productivity using meta-analysis. Agric. Ecosyst. Environ. 144, 175–187 (2011).

[b32] Steiner C. . Long term effects of manure, charcoal and mineral fertilization on crop production and fertility on a highly weathered central Amazonian upland soil. Plant Soil. 291, 275–290 (2007).

[b33] Wisnubroto E. I., Hedley M., Hina K. & Camps-Arbestain M. The use of biochar from biosolids on Waitarere sandy soils: effect on the growth of ryegrass. The New Zealand Biochar Research Centre Workshop Massey University, Palmerton North, New Zealand, 10–11 (2011).

[b34] Lehmann J. . Nutrient availability and leaching in an archaeological Anthrosol and a Ferralsol of the Central Amazon basin: fertilizer, manure and charcoal amendments. Plant Soil. 249, 343–357 (2003).

[b35] Major J., Rondon M., Molina D., Riha S. J. & Lehmann J. Maize yield and nutrition during 4 years after biochar application to a Colombian savanna oxisol. Biol. Fertil. Soils. 333, 117–128 (2010).

[b36] Uzoma K. C. . Effect of cow manure biochar on maize productivity under sandy soil condition. Soil Use Manage. 27, 205–212 (2011).

[b37] Khaliq T., Mahmood T. Kamal J. & Masood A. Effectiveness of farmyard manure, poultry manure and nitrogen for corn (*Zea mays* L.) productivity. Int. J. Agr. Biol. 6, 260–263 (2004).

[b38] Randall G. W., Vetsch J. A. & Huffman J. R. Corn production on a subsurface-drained mollisol as affected by time of nitrogen application and nitrapyrin. Agron. J. 95, 1213–1219 (2003). 10.2134/jeq2003.1764 14535319

[b39] Zhao S. C., He P., Sha Z. M., Xing S. L. & Li K. J. Evaluation of in-season nitrogen management for summer maize in North Central China. ISRN Agron. 2012, 1–9 (2011).

[b40] Karunatilake U., van Es H. M. & Schindelbeck R. R. Soil and maize response to plow and no-tillage after alfalfa-to-maize conversion on a clay loam soil in New York. Soil Till. Res. 55, 31–42 (2000).

[b41] Hatfield J. L., Sauer T. J. & Prueger J. H. Managing soils to achieve greater water use efficiency. Agron. J. 93, 271–280 (2001).

[b42] Jonard F. . Characterization of tillage effects on the spatial variation of soil properties using ground-penetrating radar and electromagnetic induction. Geoderma. 207, 310–322 (2013).

[b43] Ismail I., Blevins R. L. & Frye W. W. Long-term no-tillage effects on soil properties and continuous corn yields. Soil Sci. Soc. Am. J. 58, 193–198 (1994).

[b44] Guan D. . Tillage practices affect biomass and grain yield through regulating root growth, root-bleeding sap and nutrients uptake in summer maize. Field Crops Res. 157, 89–97 (2014).

[b45] Linn D. M. & Doran J. W. Effect of water-filled pore space on carbon dioxide and nitrous oxide production in tilled and nontilled soils. Soil Sci. Soc. Am. J. 48, 1267–1272 (1984).

[b46] Deng Q. . Corn yield and soil nitrous oxide emission under different fertilizer and soil Management: A three-year field experiment in middle Tennessee. PLoS One. 10, e0125406 (2015). 25923716 10.1371/journal.pone.0125406 PMC4414621

[b47] Aguilera E., Lassaletta L., Gattinger A. & Gimeno B. S. Managing soil carbon for climate change mitigation and adaptation in Mediterranean cropping systems: A meta-analysis. Agriculture, Ecosystems & Environment 168, 25–36 (2013).

[b48] Zhang A. . Effect of biochar amendment on maize yield and greenhouse gas emissions from a soil organic carbon poor calcareous loamy soil from Central China Plain. Plant Soil. 351, 263–275 (2012).

[b49] Martinsen V. . Farmer-led maize biochar trials: Effect on crop yield and soil nutrients under conservation farming. J. Plant Nutr. Soil Sci. 177, 681–695 (2014).

[b50] Cakir R. Effect of water stress at different development stages on vegetative and reproductive growth of corn. Field Crops Res. 89, 1–16 (2004).

[b51] Nooden L. D. Senescence in Plants. (ed. Thimann K. V.) 219–258 (Boca Raton, 1980).

[b52] Schlemmer M. R., Francis D. D., Shanahan J. F. & Schepers J. S. Remotely measuring chlorophyll content in corn leaves with differing nitrogen levels and relative water content. Agron. J. 97, 106–112 (2005).

[b53] Heichel G. H. & Musgrave R. B. Varietal differences in net photosynthesis of *Zea mays* L. Crop Sci. 9, 483–486 (1969).

[b54] Edmeades G. O. & Daynard T. B. The relationship between final yield and photosynthesis at flowering in individual maize plants. Can. J. Plant Sci. 59, 585–601 (1979).

[b55] Richards R. A. Selectable traits to increase crop photosynthesis and yield of grain crops. J. Exp. Bot. 51, 447–458 (2000). 10938853 10.1093/jexbot/51.suppl_1.447

[b56] Medrano H. . From leaf to whole-plant water use efficiency (WUE) in complex canopies: limitations of leaf WUE as a selection target. Crop J. 3, 220–228 (2015).

[b57] Troutman T. & Rose M. *A precipitation climatology for the hydrologic service area of NWSO Nashville, Tennessee*. Available at: http://www.srh.noaa.gov/ssd/techmemo/sr202.htm. (Accessed: 01/04/2016) (2009).

[b58] Mueller D. & Pop R. *Corn Field Guide - Iowa State University*. Available at : http://www.agronext.iastate.edu/corn/docs/corn-field-guide.pdf. (Data of access: 01/04/2016) (2009).

[b59] Hui D. & Jiang C. Practical Statistical Analysis System (SAS) Usage. (eds. Hui D. & Jiang C.) (Beijing, 1996).

